# Chinese childrens’ diabetes status, trends and hardship

**DOI:** 10.1186/1687-9856-2013-S1-O13

**Published:** 2013-10-03

**Authors:** Junfen Fu, Li Liang, Chunxiu Gong, Feng Xiong, Feihong Luo, Geli Liu, Pin Li, Li Liu, Ying Xin, Hui Yao, Lanwei Cui, Xing Shi, Yu Yang, Linqi Chen, Haiyan Wei

**Affiliations:** 1 Children's Hospital of Zhejiang University School of Medicine, Hangzhou, China; 2 Beijing Children’s Hospital of Capital Medical University, Beijing, China; 3 Children's Hospital of Chongqing Medical University, Yuzhong, Chongqing, China; 4 Children's Hospital of Shanghai Fudan University, Shanghai, China; 5 General Hospital of Tianjin Medical University, Tianjin, China; 6 Children's Hospital of Shanghai, Children's Hospital of Shanghai Jiaotong University, Shanghai, China; 7 Guangzhou Women and Children’s Medical Center, Guangzhou, China; 8 Shengjing Hospital of China Medical University, Shenyang, China; 9 Wuhan Children's Hospital, Wuhan, China; 10 The First Affiliated Hospital of Harbin Medical University, Harbin, China; 11 Nanjing Children's Hospital, Nanjing Medical University, Nanjing, China; 12 Children's Hospital of Jiangxi Province, Jiangxi, China; 13 Children's Hospital of Soochow University, Jiangsu, China; 14 Zhengzhou Children's Hospital, Zhenzhou, China

## 

Diabetes mellitus is now fast emerging as one of the biggest health catastrophes the world has ever witnessed. It has huge global and societal implications, particularly in developing countries such as China and India [1]. China is now bring with it potential massive increase in type 1 diabetes (2-5% per annum increases in incidence in the world's most populous countries) and childhood obesity (with its associated insulin resistance and type 2 diabetes)[2]. A nine-year prospective study on the incidence of childhood type 1 diabetes mellitus in China by the WHO DiaMond Project China Participating Center and Chinese Academy of Preventive Medicine (CAPM) showed that between 1988 and 1996, the overall incidence rate (IR) was 0.59 per 100,000 person-year. The IR was 0.52/100,000 (95% CI: 0.50-0.54) for males and 0.66/100,000 (95% CI: 0.64-0.68) for females[3]. We recently conducted a nationwide study to evaluate the state and the trend of diabetes based on hospital inpatient data from China’s 14 medical centers and pre-diabetes among obese children from October 1995 through September 2010. We found that in the past 15 years, the prevalence of Chinese childhood diabetes increased dramatically and the growth of T2DM has exceeded T1DM. T1DM has occurrence rate of 89.6% of all diabetes and is still the dominant form of diabetes in children. The prevalence of T1DM was relatively stable from the year of 1995 to 2005, but increased obviously in the recent 5 years according to the hospital records in China. The clear increasing trend from Southwest to East and North disclosed strong regional differences (T1DM from 59.76 to 80.02 and 120.45, T2DM from 2.52 to 3.77 and 15.64 (1/100,000) (p all < 0.0001). Well developed areas had a higher prevalence compared to less developed areas {T1DM: 151.51 vs. 32.2; T2DM: 15.16 vs. 1.64 and other types: 7.54 vs. 0.42 (1/100,000)}. An important finding in this study is that the prevalence of childhood T2DM in China doubled from 4.1/100,000 in the first 5 years to 10.0/100,000 in the recent 5 years, which was 7.44 % of total diabetics. Though the ratio is still lower than that of America (8%-46%)[4] , the trend is clear and the consequences are serious because China has the largest population in the world. Another important finding in this study showed obese children are potential pools of T2DM. Of the 3153 obese children, 18.24% had IFG alone, 5.99% had IGT, 4% had combined IFG and IGT.

## References

[B1] Meetoo D McGovern P Safadi R An epidemiological overview of diabetes across the world Br J Nurs 2007 2013 16 1002 7 18026039 10.12968/bjon.2007.16.16.27079

[B2] Daneman D State of the world's children with diabetes Pediatr Diabetes 2009 2013 2 120 6 Epub 2008 Oct 7 10.1111/j.1399-5448.2008.00479.x 19175511

[B3] Li XH Li TL Yang Z Liu ZY Wei YD Jin SX Hong C Qin RL Li YQ Dorman JS Laporte RE Wang KA A nine-year prospective study on the incidence of childhood type 1 diabetes mellitus in China Biomed Environ Sci 2000 2013 4 263 70 11351859

[B4] Mohamadi A Cooke DW Type 2 diabetes mellitus in children and adolescents Adolesc Med State Art Rev 2010 2013 1 103 119 20568558

